# Maximum Augmented Empirical Likelihood Estimation of Categorical Marginal Models for Large Sparse Contingency Tables

**DOI:** 10.1007/s11336-023-09932-7

**Published:** 2023-09-26

**Authors:** L. Andries van der Ark, Wicher P. Bergsma, Letty Koopman

**Affiliations:** 1 Research Institute of Child Development and Education, University of Amsterdam, P.O. Box 15776, 1001 NG Amsterdam, The Netherlands; 2 THE London School of Economics AND POLITICAL SCIENCE, London, UK

**Keywords:** categorical marginal model, Cronbach’s alpha, large categorical data sets, marginal homogeneity, maximum empirical likelihood estimation, maximum likelihood estimation, scalability coefficients

## Abstract

Categorical marginal models (CMMs) are flexible tools for modelling dependent or clustered categorical data, when the dependencies themselves are not of interest. A major limitation of maximum likelihood (ML) estimation of CMMs is that the size of the contingency table increases exponentially with the number of variables, so even for a moderate number of variables, say between 10 and 20, ML estimation can become computationally infeasible. An alternative method, which retains the optimal asymptotic efficiency of ML, is maximum empirical likelihood (MEL) estimation. However, we show that MEL tends to break down for large, sparse contingency tables. As a solution, we propose a new method, which we call maximum augmented empirical likelihood (MAEL) estimation and which involves augmentation of the empirical likelihood support with a number of well-chosen cells. Simulation results show good finite sample performance for very large contingency tables.

Categorical marginal models (CMMs; Bergsma et al., 2009; also see, e.g., Bergsma, 1997; Bergsma & Rudas, 2002; Bartolucci et al., 2007; Colombi & Forcina, 2001; Evans & Forcina, 2013; Lang & Agresti, 1994; Lang, 1996; Molenberghs & Lesaffre, 1999; Rudas & Bergsma, 2023) are flexible tools to model location, spread, and association in dependent or clustered categorical data, when the dependence itself is not of interest. CMMs require data in a table format for input; that is, for a dataset with *N* respondents and *J* categorical variables, CMMs require a (vectorized) *J*-variate contingency table, where each cell corresponds to a response pattern, and the frequencies within the cells represent the observed frequencies of each response pattern. The only assumption of the CMMs under consideration is that the cell frequencies in the contingency table follow a multinomial distribution, rendering a very flexible method.

CMMs can be a valuable psychometric tool since they allow for null-hypothesis significance testing (NHST) of complex coefficients without the need to specify a parametric model or impose additional assumptions. In Psychometrics, NHTS often occurs under the assumption of a parametric model. For example, testing measurement invariance across several groups is typically done under a structural equation model (e.g., Cheung & Rensvold, 2002). However, rather than testing $H_0$ (the null-hypothesis of interest), we implicitly test $H_0^*$: $H_0$ plus the assumption that the structural equation model fits the data. Rejecting $H_0^*$ does not provide information about $H_0$ because $H_0^*$ should be rejected either when $H_0$ is false or when the structural equation model does not fit the data (cf. Jorgensen et al., 2017). In other fields of psychometrics (e.g., nonparametric modeling, classical test theory) and applied statistics, there is no comprehensive parametric modeling framework. In such situations, it becomes particularly valuable if the assumptions required for NHST are easily satisfied, ensuring that the null hypothesis of interest is not excessively confounded by data failing to meet the assumptions, thus maintaining a close approximation between $H_0^*$ and $H_0$. The CMM assumption that cell frequencies follow a multinomial distribution is very lenient, implying that every response pattern should, in principle, be observable.

The process of relaxing assumptions for NHST can be a time-consuming endeavor spanning several years. For instance, in the case of NHST for Cronbach’s alpha, there exists a history of research papers progressively relaxing the required assumptions: Feldt derived tests for three types of null-hypothesis on Cronbach’s alpha: alpha equals some criterion value (Feldt, 1969), alpha is equal across groups (Feldt, 1965), and alpha is equal across different measurements (Feldt 1980). Feldt assumed that alpha asymptotically follows an F distribution. This assumptions was subsequently relaxed by Van Zyl et al. (2000), who derived a distribution without restricting the covariances, Maydeu-Olivares et al. (2007) who relaxed the assumptions of Feldt’s first hypothesis by deriving asymptotically distribution-free interval estimates for alpha, Maydeu-Olivares et al. (2010) who proposed testing Feldt’s hypotheses in a structural equation modeling framework, and ultimately, Kuijpers et al. (2013), who proposed using CMMs for testing Feldt’s hypotheses. Each successive paper demonstrated significant enhancements in the properties of NHST for Cronbach’s alpha when compared to its predecessors.

In some cases, no hypothesis tests are available leaving CMMs as a possible option to derive hypothesis tests. For example, Van der Ark et al. (2008) used CMMs for developing NHST for Mokken’s (1971) scalability coefficients, which allows testing scalability coefficients for item pairs, individual items, and scales across groups and across measurement occasions. Finally, we would like to note that CMMs can be used in conjunction with latent variables models, although this needs further development. We refer to Bergsma et al. (2009), for other applications of CMMs, and Bergsma et al. (2009, 2013) who introduced CMMs with latent variables.

CMMs can be estimated using the maximum likelihood (ML) method, which has many favorable properties, including asymptotic efficiency. A serious limitation of the ML method is that for large contingency tables estimation is infeasible, as ML requires the computation of an expected frequency for each cell in the contingency table. This *curse of dimensionality* may be an important reason why CMMs have failed to become popular in psychometrics. Most psychological and educational tests consist of many variables (usually referred to as items) yielding an extremely large number of possible response patterns and, therefore, extremely large contingency tables. For example, Raven’s Advanced Progressive Matrices (Raven et al., 2003), measuring general intelligence, consists of 48 binary items, which yields a contingency table of $2^{48} \approx 2.81 \times 10^{14}$ cells; and the personality inventory NEO-PI-R (Costa & McCrae 2008), measuring five personality traits, consists of 48 five-category items per trait, which yields a contingency table of $5^{5 \times 48} \approx 5.66 \times 10^{167}$ cells. Lloyd (2000) estimated that if every particle in the universe could be used as part of a huge computer, it could store approximately $10^{90}$ bits. Hence, for contingency tables based on psychological and educational tests, the required computer capacity easily exceeds the ultimate physical limits of computation, whereas The ML estimation procedure to estimate CMMs implemented in the R-package cmm (Bergsma & Van der Ark, 2023) cannot handle more than a few million cells.

In this paper, we give a new adaptation to the ML estimation procedure to solve the above problem. Although there are alternative estimation procedures that may be used to estimate CMMs, we preferred to stay within a ML-framework as ML guarantees asymptotic efficiency, whereas alternatives estimation methods for contingency tables, such as generalizing estimation equations (GEE’s, e.g., Qaqish & Liang, 1992), and composite likelihood (e.g., Varin et al., 2011) are not, and weighted least squares (Grizzle et al., 1969; a.k.a the GSK-method) is sensitive to sparsity in the marginal distribution (cf. Rudas & Bergsma, 2023). In addition, an adaptation of the ML approach is easy to fit in the existing software.

Initially, we considered the *empirical likelihood* method (Owen, 2001, Qin & Lawless, 1994), a data-driven, nonparametric estimation method. The core idea behind the empirical likelihood method is to construct a likelihood function directly from the observed data, without assuming any specific underlying probability distribution; that is, given vector valued data $\textbf{x}_1,\ldots ,\textbf{x}_N$, an empirical likelihood is the likelihood of a probability distribution with support $\{\textbf{x}_1,\ldots ,\textbf{x}_N\}$ (Owen, 2001). In the context of CMMs, the empirical likelihood method involves constructing the likelihood solely from cells with nonzero frequencies, while regarding cells with zero frequency as structural zeroes and setting their estimated probability to zero. Given that the number of cells with nonzero frequencies cannot exceed the sample size, and in the case of psychological and educational test data, the sample size rarely exceeds 10,000, the empirical likelihood method serves as a computationally feasible alternative to ML. We abbreviate the method of maximizing the empirical likelihood subject to model constraints by MEL.

Unfortunately, the support $\{\textbf{x}_1,\ldots ,\textbf{x}_N\}$ belonging to the empirical likelihood may be too small (i) to estimate the parameters of a CMM, or, even if this can be done, (ii) to estimate the asymptotic covariance matrix of the ML estimators of the parameters of the CMM. We will refer to these two problems as the *first- and second-order estimation problems*, respectively (see Appendix A for more details). The first problem has also been called the *empty set problem* (Grendár & Judge, 2009). As far as we are aware, the second problem has not yet been described in the literature. The solution to these problems which we propose in this paper is to augment the empirical likelihood support with a number of well-chosen points, and we will refer to the method of maximizing the resulting empirical likelihood as *maximum augmented empirical likelihood* (MAEL). Note that as the sample size goes to infinity, assuming no structural zeroes, the probability that all cells in a contingency table will have a positive count will go to 1, so for categorical data MEL, MAEL and ML are asymptotically equivalent.

The reason why MEL and MAEL estimators work asymptotically (as $N \rightarrow \infty$) is because they are with probability tending to 1 equivalent to ML estimator. That justifies testing goodness of fit and making inferences for parameters in same ways as we would do with ML. Two related methods, called *adjusted empirical likelihood*, Chen et al. (2008) and *balanced augmented empirical likelihood* (Emerson & Owen, 2009; also see Nguyen et al., 2015, Xia & Liu, 2019) have been considered for continuous data. These methods augment the *data set* with one or two additional observations. In contrast, our methodology consists of only augmenting the *support* of distributions corresponding to the empirical likelihood with additional points, but without adding any observations to the data.

The remainder of the paper is organized as follows. In Sect. 1, we give a brief overview of and notation for CMMs. In Sect. 2, we describe ML and MEL estimation for CMMs and introduce MAEL estimation. In Sect. 3, we present two simulation studies. Study 1 compares the convergence rate and computation time of ML, MEL, and MAEL estimation for small contingency tables, and Study 2 investigates the Type I error rate of CMMs using MAEL estimation for small and large contingency tables, and bias and variance of the model parameters. In Sect. 4, we briefly discuss the advantages and disadvantages of MAEL estimation in relation to other, non-likelihood-based estimation procedures. In Appendix A, we describe the first- and second-order estimation problems in some generality, whereas Appendix B gives details of the estimation algorithm used.

## CMMs

Consider the categorical variables $X_1, \dots , X_j, \dots , X_J$ with $X_j \in \{0, \dots g_j\}$. Let $\textbf{x}_1,\ldots ,\textbf{x}_i,$ $\ldots ,\textbf{x}_N$ be i.i.d. data points, where each $\textbf{x}_i=(x_{i1},\ldots ,x_{iJ})$ consists of the scores of the *i*th respondent on the variables $X_1, \dots , X_J$. The data can be collected in a *J*-way contingency table of observed frequencies with $L = \prod _{j=1}^J g_j$ cells. The observed frequency of the response pattern $(x_1, \ldots , x_J)$ on variables $(X_1, \ldots , X_J)$ is denoted by $n\hspace{-.5pt}{{^{X_1,}_{x_1,}}}\hspace{-.5pt}{{^{\dots ,}_{\dots ,}}}\hspace{-.5pt}{{^{X_J}_{x_J}}}$. The observed frequencies in the contingency table are collected in an $L\times 1$ vector $\textbf{n}$, arranged in lexicographical order; that is, the digit in the last row of the corresponding response pattern changes fastest and the digit in the first row changes slowest. As an example, Eq. 1 shows the vector $\textbf{n}$ containing the observed frequencies of the response patterns pertaining to the scores of $N = 130$ respondents on $J = 3$ binary variables, *a*, *b*, and *c*:

1 $$\begin{aligned} \textbf{n} = \left( \begin{array}{c} n^{abc}_{000}\\ n^{abc}_{001}\\ n^{abc}_{010}\\ n^{abc}_{011}\\ n^{abc}_{100}\\ n^{abc}_{101}\\ n^{abc}_{110}\\ n^{abc}_{111}\\ \end{array} \right) = \left( \begin{array}{c} 20\\ 15\\ 10\\ 15\\ 0\\ 15\\ 25\\ 30\\ \end{array} \right) . \end{aligned}$$

If it is clear which variables are involved, then the superscript may be omitted. Marginal frequencies are denoted by removing the appropriate variable(s) from the subscript and score(s) from the superscript. In some formulas, the subscript *i* in $n_i$ is used as an index. For example, $\sum _i n_i$ means the sum over all elements of $\textbf{n}$.

The probability that a randomly drawn respondent has response pattern $x_1, \dots , x_J$ given that the CMM of interest is true, is denoted by $\pi \hspace{-.5pt}{{^{X_1,}_{x_1,}}}\hspace{-.5pt}{{^{\dots ,}_{\dots ,}}}\hspace{-.5pt}{{^{X_J}_{x_J}}}$. Assuming a fixed sample size *N*, let $m\hspace{-.5pt}{{^{X_1,}_{x_1,}}}\hspace{-.5pt}{{^{\dots ,}_{\dots ,}}}\hspace{-.5pt}{{^{X_J}_{x_J}}}$ be the expected frequency satisfying $m\hspace{-.5pt}{{^{X_1,}_{x_1,}}}\hspace{-.5pt}{{^{\dots ,}_{\dots ,}}}\hspace{-.5pt}{{^{X_J}_{x_J}}} = N \times \pi \hspace{-.5pt}{{^{X_1,}_{x_1,}}}\hspace{-.5pt}{{^{\dots ,}_{\dots ,}}}\hspace{-.5pt}{{^{X_J}_{x_J}}}$. The expected frequencies and probabilities are collected in vectors $\textbf{m}$, and ${{\varvec{\uppi }}}$, respectively, in the same manner as the observed frequencies were collected in $\textbf{n}$. ML estimates of $\textbf{m}$ and ${{\varvec{\uppi }}}$ are denoted by $\widehat{\textbf{m}}$ and $\widehat{{{\varvec{\uppi }}}}$, respectively. Without any constraints imposed upon the data, $\widehat{\textbf{m}} = \textbf{n}$ and $\widehat{{{\varvec{\uppi }}}} = \textbf{n}/N$.

Let $\textbf{A}$ be a matrix of zeroes and ones, so that $\textbf{A}^\textrm{T}\textbf{m}$ consists of the relevant marginals from the contingency table. A CMM is defined by constraints of the form

2 $$\begin{aligned} \textbf{f}(\textbf{A}^\textrm{T}\textbf{m}) = \textbf{Z} {\varvec{\upbeta }}, \end{aligned}$$

where $\textbf{f}$ is an appropriate function, $\textbf{Z}$ is a design matrix of full column rank, and ${\varvec{\upbeta }}$ is a vector of parameters. For estimation purposes, parameter ${\varvec{\upbeta }}$ is eliminated from the equation as follows. Let $\textbf{B}$ be the orthogonal complement of the column space spanned by the columns of $\textbf{Z}$ (i.e., $\textbf{B}^\textrm{T}\textbf{Z} = \textbf{0}$ and the concatenated matrix $(\textbf{B}\,\,\, \textbf{Z})$ is square and non-singular). By pre-multiplying both sides of Eq. 2 by $\textbf{B}^\textrm{T}$, the CMM is written as a set of constraints:

3 $$\begin{aligned} \textbf{B}^\textrm{T} \textbf{f}(\textbf{A}^\textrm{T}\textbf{m}) = \textbf{B}^\textrm{T} \textbf{Z} {\varvec{\upbeta }}= \textbf{0}. \end{aligned}$$

Note that parameter ${\varvec{\upbeta }}$ can be obtained from Eq. 2 by

4 $$\begin{aligned} {\varvec{\upbeta }}= (\textbf{Z}^\textrm{T} \textbf{Z})^{-1} \textbf{Z}^\textrm{T} \textbf{f}(\textbf{A}^\textrm{T}\textbf{m}). \end{aligned}$$

The constraint formulation $\textbf{B}^\textrm{T} \textbf{f}(\textbf{A}^\textrm{T}\textbf{m}) = \textbf{0}$ (cf. Eq. 3) is computationally convenient since it allows the Lagrange multiplier technique to be used, and asymptotic theory has been developed using this formulation (Aitchison & Silvey, 1958, Lang, 2005). In addition, the parameter formulation $\textbf{f}(\textbf{A}^\textrm{T}\textbf{m}) = \textbf{Z} {\varvec{\upbeta }}$ (Eq. 2) is not possible if $\textbf{B}^\textrm{T}$ is of full column rank because $\textbf{Z}$, the orthogonal complement of $\textbf{B}$, does not exist. Therefore, the parameter formulation of CMMs will be disregarded from here on.

For notational convenience, we can replace $\textbf{B}^\textrm{T} \textbf{f}(\textbf{A}^\textrm{T}\textbf{m})$ by $\textbf{g}(\textbf{m})$. So, the shortest notation for a CMM is

5 $$\begin{aligned} \textbf{g}(\textbf{m}) = \textbf{0}. \end{aligned}$$

Let *D* be the number of constraints in Eq. 5; that is, the length of vector $\textbf{g}(\textbf{m})$. The fit of the CMM can be investigated by comparing $\textbf{n}$ and the ML estimate under the model, $\widehat{\textbf{m}}$, using a likelihood ratio test statistic ($G^2$) or Pearson’s Chi-square test statistic ($X^2$), which have an asymptotic Chi-square distribution with *D* degrees of freedom if the model is true. Example 1 shows a simple CMM following the build up in Eqs. 2, 3, 4, and 5, whereas Example 2 shows a CMM that has been used in psychometrics.

### Example 1

Consider $\textbf{n}$ in Eq. 1. Suppose that we want to fit the CMM that prescribes marginal homogeneity: $m^{a}_1 = m^{b}_1 = m^{c}_1$ (and consequently, $m^{a}_0 = m^{b}_0 = m^{c}_0$). First, pre-multiplying $\textbf{m}$ by design matrix $\textbf{A}^\textrm{T}$ (Eq. 2) yields the required margins; that is,

6 $$\begin{aligned} \textbf{A}^\textrm{T}\textbf{m} = \left( \begin{array}{rrrrrrrr} 0 &{} 0 &{} 0 &{} 0 &{} 1 &{} 1 &{} 1 &{} 1\\ 0 &{} 0 &{} 1 &{} 1 &{} 0 &{} 0 &{} 1 &{} 1\\ 0 &{} 1 &{} 0 &{} 1 &{} 0 &{} 1 &{} 0 &{} 1\\ \end{array} \right) \cdot \left( \begin{array}{r} m^{abc}_{000}\\ m^{abc}_{001}\\ m^{abc}_{010}\\ m^{abc}_{011}\\ m^{abc}_{100}\\ m^{abc}_{101}\\ m^{abc}_{110}\\ m^{abc}_{111}\\ \end{array} \right) = \left( \begin{array}{r} m^{a}_{1}\\ m^{b}_{1}\\ m^{c}_{1} \end{array} \right) . \end{aligned}$$

Function $\textbf{f}$ (Eq. 2) is the identity function, so $\textbf{f}(\textbf{A}^\textrm{T}\textbf{m}) = \textbf{A}^\textrm{T}\textbf{m} = (m_1^a~m_1^b~m_1^c)^\textrm{T}$. To write the CMM as a set of constraints, $\textbf{f}(\textbf{A}^\textrm{T}\textbf{m})$ is pre-multiplied by constraint matrix $\textbf{B}^\textrm{T}$ (cf. Eq. 3, left-hand side), and set to zero, yielding

7 $$\begin{aligned} \textbf{B}^\textrm{T} \textbf{f}(\textbf{A}^\textrm{T}\textbf{m}) = \left( \begin{array}{rrr} 1 &{} -1 &{} 0 \\ 0 &{} 1 &{} -1 \end{array} \right) \cdot \left( \begin{array}{c} m^{a}_{1}\\ m^{b}_{1}\\ m^{c}_{1} \end{array} \right) = \left( \begin{array}{c} m^{a}_{1} - m^{b}_{1}\\ m^{b}_{1} - m^{c}_{1} \end{array} \right) = \left( \begin{array}{c} 0\\ 0 \end{array} \right) . \end{aligned}$$

As the $3\times 1$ column vector $\textbf{Z} = \left( \frac{1}{\sqrt{3}}~\frac{1}{\sqrt{3}}~ \frac{1}{\sqrt{3}}\right)$ is the orthogonal complement of $\textbf{B}$, with $(\textbf{Z}^\textrm{T}\textbf{Z})^{-1} = 1$, parameter $\beta$ (which in this case is 1-dimensional) can be obtained by Eq. 4; that is,

8 $$\begin{aligned} \beta = (\textbf{Z}^\textrm{T} \textbf{Z})^{-1} \textbf{Z}^\textrm{T} \textbf{f}(\textbf{A}^\textrm{T}\textbf{m}) = 1 \cdot \left( \begin{array}{ccc} \frac{1}{\sqrt{3}}&\frac{1}{\sqrt{3}}&\frac{1}{\sqrt{3}} \end{array} \right) \left( \begin{array}{c} m^{a}_{1}\\ m^{b}_{1}\\ m^{c}_{1} \end{array} \right) = \frac{m^{a}_{1} + m^{b}_{1} + m^{c}_{1}}{\sqrt{3}}. \end{aligned}$$

Conventional short notation $\textbf{g}(\textbf{m}) =\textbf{0}$ (Eq. 5) is obtained by letting $\textbf{g}(\textbf{m}) = \textbf{B}^\textrm{T}\textbf{f}(\textbf{A}^\textrm{T} \textbf{m})$; that is,

9 $$\begin{aligned} \textbf{g}(\textbf{m}) = \left( \begin{array}{rrr} 1 &{} -1 &{} 0 \\ 0 &{} 1 &{} -1 \end{array} \right) \cdot \left( \begin{array}{rrrrrrrr} 0 &{} 0 &{} 0 &{} 0 &{} 1 &{} 1 &{} 1 &{} 1\\ 0 &{} 0 &{} 1 &{} 1 &{} 0 &{} 0 &{} 1 &{} 1\\ 0 &{} 1 &{} 0 &{} 1 &{} 0 &{} 1 &{} 0 &{} 1\\ \end{array} \right) \cdot&\textbf{m} \nonumber \\ = \left( \begin{array}{rrrrrrrr} 0 &{} 0 &{}-1 &{}-1 &{} 1 &{} 1 &{} 0 &{} 0\\ 0 &{}-1 &{} 0 &{}-1 &{} 1 &{} 0 &{} 1 &{} 0\\ \end{array} \right)&\left( \begin{array}{r} m^{abc}_{000}\\ m^{abc}_{001}\\ m^{abc}_{010}\\ m^{abc}_{011}\\ m^{abc}_{100}\\ m^{abc}_{101}\\ m^{abc}_{110}\\ m^{abc}_{111}\\ \end{array} \right) = \left( \begin{array}{r} 0\\ 0\\ \end{array} \right) . \end{aligned}$$

The vector of expected frequencies that is closest (in an ML sense) to $\textbf{n}$ (Eq. 1) and meets the requirement of Eq. 9 is

10 $$\begin{aligned} \widehat{\textbf{m}} = \left( \begin{array}{r} 20.000\\ 14.397\\ 8.060\\ 11.695\\ 0.000\\ 19.755\\ 26.092\\ 30.000\\ \end{array} \right) . \end{aligned}$$

Comparing $\textbf{n}{}$ given in Eq. 1 and $\widehat{\textbf{m}}{}$ given in Eq. 10 yields $G^2 = 2.6107$ (df = 2, $p=.2711).{ UsinganominalTypeIerrorrateof}\alpha =.05$, the hypothesis of marginal homogeneity should not be rejected.

### Example 2

Item-scalability coefficient $H_j (j=1,\dots ,J$) is used in Mokken scale analysis (e.g., Mokken, 1971; Sijtsma & Van der Ark, 2017) and expresses the strength of the relationship between item $j$ and the other items in the test, comparable with a regression coefficient in a regression model. One of the criteria of a *Mokken scale* is that all coefficients $H_j{ aregreaterthansomelowerbound}c.{ Thelowerboundthatisusedasadefaultis}c =0.30$ (Sijtsma & Molenaar 2002). Hence, a relevant question is whether all $H_j >0.30.{ Coefficients}H_j{ arenotindependentfromeachother},\,{ andCMMscanbeusedtocontrolforthisnuisancedependenceandtestallcoefficientssimultaneously}.{ Undertheassumptionthattheitemsarenumberedinanascendingorderoftheirprobabilityofansweringtheitemcorrectly}({ i}.{ e}.,\,{ item}1{ istheleastpopularormostdifficultitem},\,{ item}J$ the most popular or least difficult item), item-scalability coefficients $H_j,\,j = 1, \dots , J$ for dichotomous items (Mokken, 1971, p. 151) are defined as

11 $$\begin{aligned} H_{j} = 1 - \frac{N \left( \sum \nolimits _{i = 1}^{j-1} m^{ij}_{01} + \sum \nolimits _{i = j+1} ^J m^{ji}_{01}\right) }{\sum \nolimits _{i = 1}^{j-1} m^i_0 m^j_1 + \sum \nolimits _{i = j+1} ^J m^j_0 m^i_1}. \end{aligned}$$

Consider the observed frequencies in Eq. 1. Let $\textbf{H} = (H_a, H_b, H_c)$ be a vector containing the item-scalability coefficients of items $a,\,b,\,{ and}c$. Equation 11 shows that $\textbf{H}{} { isafunctionof}\textbf{m}.{ Theconstraints}\textbf{H} - (0.3,0.3,0.3)^\textrm{T} = \textbf{0}{}$ defines a CMM (Eq. 5); we refer to Van der Ark et al. (2008) for computational details.

The sample values of $H_j$ for the vector of observed frequencies in Eq. 1 are $\widehat{H}_a =0.231,\,\widehat{H}_b =0.164,\,{ and}\widehat{H}_c =0.055$. Fitting the CMM that all item-scalability coefficients equal 0.3 to the data in Eq. 1 yields $G^2 = 14.84 (df = 3, p =0.0023).{ UsinganominalTypeIerrorrateof}\alpha =0.05,\,{ thehypothesis}\textbf{H} = (0.3,0.3,0.3)^T$ should be rejected.

## Estimation of CMMs

### ML and MEL Estimation

Assuming that the frequency vector $\textbf{n}{}$ follows a multinomial distribution, the likelihood function is

12 $$\begin{aligned} \mathcal {L}(\textbf{m}|\textbf{n}) = \frac{N!}{\prod _{i=1}^L n_i!} \prod _{i=1}^L \left( \frac{m_i}{N} \right) ^{n_i} \propto \prod _{i=1}^L m_i^{n_i}. \end{aligned}$$

The maximum likelihood estimate $\widehat{\textbf{m}}{} { maximizes}{{\mathcal {L}}}(\textbf{m}|\textbf{n})$ subject to the *model constraint*

13 $$\begin{aligned} \textbf{g}(\textbf{m}) = \textbf{0} \end{aligned}$$

and the *multinomial constraint*

14 $$\begin{aligned} \sum _i m_i = N = \sum _i n_i. \end{aligned}$$

In Appendix B, an algorithm for finding $\widehat{\textbf{m}}{} { isgiven}.{ Formultinomialdistributions},\,{ MELestimationissimilartoMLestimation},\,{ withthedifferencethatallcellsforwhich}n_i=0{ aretreatedasstructuralzeros}.{ TheMELestimateof}\textbf{m}{} { maximizes}\mathcal {L}(\textbf{m}|\textbf{n})$ subject to Eqs. 13 and 14 and the *structural-zero constraint*

15 $$\begin{aligned} m_i = 0 \text{ if } n_i = 0. \end{aligned}$$

MEL estimation can be done using the same algorithm as ML estimation because the cells $i{ forwhich}n_i=0{ cansimplybeleftoutoftheestimationprocedure}.{ ForMELestimation},\,{ fewercellsneedtobeestimated},\,{ whichmakestheprocedurefasterandmoresuitableforlargecontingencytablesthanMLestimation}.{ Ingeneral},\,{ asuperscriptedasteriskindicatesthatthecells}i{ forwhich}n_i=0{ areleftout};\,{ thatis},\,L^*{ isthenumberofcellsforwhich}n_i>0,\,\textbf{n}^*{ isthevectoroflength}L^*{ ofnonzeroobservedfrequencies}({ i}.{ e}.,\,\textbf{n}^*{ isthevectorcontainingthose}n_i{ thataregreaterthanzero}).{ Thecorrespondingexpectedfrequenciesandexpectedprobabilitiesaredenoted}\textbf{m}^*{ and}{{\varvec{\uppi }}}^*,\,{ respectively},\,{ and}\textbf{g}^*(\textbf{m}^*){ equals}\textbf{g}(\textbf{m}){ withtheelementsof}\textbf{m}$ corresponding to zero observed cells set to zero. Example 3 shows an illustration of MEL estimation.

#### Example 3

This example illustrates MEL estimation of the CMM in Example 1. For the vector of observed frequencies in Eq. 1,

16 $$\begin{aligned} \textbf{n}^* = \left( \begin{array}{c} n^{abc}_{000}\\ n^{abc}_{001}\\ n^{abc}_{010}\\ n^{abc}_{011}\\ n^{abc}_{101}\\ n^{abc}_{110}\\ n^{abc}_{111}\\ \end{array} \right) = \left( \begin{array}{c} 20\\ 15\\ 10\\ 15\\ 15\\ 25\\ 30\\ \end{array} \right) . \end{aligned}$$

In Eq. 16, $n^{abc}_{100}{} { hasbeenomitted},\,{ whichimpliesthat}m^{abc}_{100}{}$ is fixed to zero, and not considered in the estimation procedure. The CMM in Eq. 9 under MEL reduces to

17 $$\begin{aligned} \textbf{g}^*(\textbf{m}^*) = \left( \begin{array}{rrrrrrr} 0 &{} 0 &{}-1 &{}-1 &{} 1 &{} 0 &{} 0\\ 0 &{}-1 &{} 0 &{}-1 &{} 0 &{} 1 &{} 0\\ \end{array} \right) \left( \begin{array}{c} m^{abc}_{000}\\ m^{abc}_{001}\\ m^{abc}_{010}\\ m^{abc}_{011}\\ m^{abc}_{101}\\ m^{abc}_{110}\\ m^{abc}_{111}\\ \end{array} \right) = \left( \begin{array}{r} 0\\ 0\\ \end{array} \right) . \end{aligned}$$

Comparing $\textbf{n}^*$ given in Eq. 16 and

$$\begin{aligned} \widehat{\textbf{m}}^* = \left( \begin{array}{r} 20.000\\ 14.397\\ 8.060\\ 11.695\\ 19.755\\ 26.092\\ 30.000\\ \end{array} \right) . \end{aligned}$$

yields $G^2 = 2.611 (df = 2, p=0.271$). In this case, ML estimation (see Example 1) and MEL estimation provide identical expected frequencies and model fit, but this is not true in general.

### The First- and Second-Order Estimation Problems for CMMs

Unfortunately, the support $\{\textbf{x}_1,\ldots ,\textbf{x}_N\}$ belonging to the empirical likelihood may be too small for the CMM to be estimated and to do inference. We identify two problems, which are described more formally and in some more generality in Appendix A. We say that the *first-order estimation problem* occurs if the equation $\textbf{g}^*(\textbf{m}^*)=\textbf{0}$ does not have any solutions. This is also known as the *empty set problem* (Grendár & Judge, 2009). The *second-order estimation problem* occurs if the empirical likelihood support is too small to be able to estimate the covariance matrix of the estimated marginal parameters. Occurrence of the first-order problem implies occurrence of the second-order problem, and absence of the second-order problem implies absence of the first-order problem. If the second-order problem occurs, inference for the model is problematic. The first- and second-order estimation problems can occur for MEL estimation with sparse observed contingency tables, as illustrated next.

#### Example 4

Consider a $2\times 2$ contingency table and let

$$\begin{aligned} \textbf{g}(\textbf{m})=(m_{0+}-m_{1+})-(m_{+0}-m_{+1}) = \left( \begin{array}{cccc} 0&1&-1&0 \end{array} \right) \left( \begin{array}{c} m_{00}\\ m_{01}\\ m_{10}\\ m_{11}\\ \end{array} \right) = 0. \end{aligned}$$

Suppose we observe

$$\begin{aligned} \left( \begin{array}{c} n_{00}\\ n_{01}\\ n_{10}\\ n_{11}\\ \end{array} \right) = \left( \begin{array}{c} 0\\ 1\\ 0\\ 0\\ \end{array} \right) . \end{aligned}$$

Then, it can be verified that $\textbf{g}^*(\textbf{m}^*)= m_{01} \times 1 = 0$ does not have any solutions; that is, the first-order estimation problem (or empty set problem) occurs, and hence so does the second-order one. If, on the other hand, we observed

$$\begin{aligned} \left( \begin{array}{c} n_{00}\\ n_{01}\\ n_{10}\\ n_{11}\\ \end{array} \right) = \left( \begin{array}{c} 1\\ 0\\ 0\\ 1\\ \end{array} \right) , \end{aligned}$$

then the first-order problem does not occur. Assuming Poisson sampling for simplicity, we have ${ \text{ var }}(\textbf{g}(\textbf{n})-\textbf{g}(\textbf{m}))=4m_{01}+4m_{10}$. Under empirical likelihood, this is zero; that is, the variance of the marginal parameter cannot be estimated, and the second-order problem occurs.

#### Example 5

Consider dichotomous variables $X_1$ and $X_2$, and let the CMM be $H_1 =0.3$. Let $\textbf{n} = (n_{00}, n_{01}, n_{10}, n_{11})^\textrm{T} = (30,0,30,30)^\textrm{T}$, hence $\textbf{n}^* = (n_{00}, n_{10}, n_{11})^\textrm{T} = (30,30,30)^\textrm{T}$. It follows that $\overline{X}_1 = \frac{2}{3}$ and $\overline{X}_2 = \frac{1}{3}$. Under the assumption that $E(X_1) > E(X_2)$, Eq. 11 reduces to

18 $$\begin{aligned} H_1 = H_2 = 1- \frac{N \times m_{01}}{m_0 \times m_1}. \end{aligned}$$

Frequency $n_{01}$ is not observed, so due to the structural-zero constraint (Eq. 15), MEL estimation produces $\widehat{m}_{01} = 0$ by definition. As a result, the ratio on the right-hand side of Eq. 18 equals zero, and $H_1 = H_2 = 1$. Hence, there exists no $\textbf{m}^*$ satisfying $H_1 =0.3$.

### MAEL Estimation

A solution to the first- and second-order estimation problems is obtained by augmenting the empirical likelihood support with a number of support cells, which we call maximum augmented empirical likelihood (MAEL) estimation. The question arises which cells to add. For CMMs, there is a fairly natural choice, in particular, suppose the order *k* marginal distributions are of interest for a particular CMM. Then clearly, to avoid the first-order estimation problem, the support must contain for every marginal cell at least one cell in the contingency table contributing to it. Hence, this is the least augmentation that should be done for the empirical likelihood support. To avoid the second-order estimation problem, note that the covariance between observed marginals is a function of higher-order marginals, for example,

$$\begin{aligned}{} & {} { \text{ cov }}(n_{i++},n_{+j+}) = m_{ij+} - m_{i++}m_{+j+}/N, \\{} & {} \quad { \text{ cov }}(n_{ij++},n_{+kl+}) = \delta _{jk}m_{ijl+} - m_{ij++}m_{+kl+}/N \end{aligned}$$

or

$$\begin{aligned} { \text{ cov }}(n_{+ij+++},n_{++++kl}) = m_{+ij+kl} - m_{+ij+++}m_{++++kl}/N \end{aligned}$$

where a plus in the subscript denotes summation over that subscript. If the relevant higher-order marginals are estimable, the second-order estimation problem can typically be avoided.

If the second-order estimation problem occurs, it can be resolved by augmenting the empirical likelihood support so that each of the relevant higher-order marginals has one or more cells contributing to it. We found that the methodology is not affected much by which cells were chosen. In practice, we randomly added cells, which gave good results.

The notation is as follows. For ML estimation, all *L* cells of $\textbf{n}$ are considered, and for MEL estimation, only the $L^*$ cells with a positive observed count, collected in $\textbf{n}^*$, are considered. MAEL can be regarded as an intermediate estimation method, considering the $L^*$ cells with a positive observed count plus a number of cells with zero observed count to avoid the first- and second-order estimation problems. Let $L^\dagger$ be such that $L^*\le L^\dagger \le L$, and let $\textbf{n}^\dagger$, $\textbf{m}^\dagger$, and ${{\varvec{\uppi }}}^\dagger$ denote the augmented vector of observed frequencies, expected frequencies, and probabilities, respectively.

Example 6 explores some possibilities to augment the empirical likelihood support for a small example, illustrating that the fit of a CMM decreases dramatically when too few cells are added to $\textbf{n}^*$.

#### Example 6

Suppose that

19 $$\begin{aligned} \textbf{n} = \left( \begin{array}{c} n^{abc}_{000}\\ n^{abc}_{001}\\ n^{abc}_{010}\\ n^{abc}_{011}\\ n^{abc}_{100}\\ n^{abc}_{101}\\ n^{abc}_{110}\\ n^{abc}_{111}\\ \end{array} \right) = \left( \begin{array}{r} 0\\ 0\\ 0\\ 0\\ 65\\ 0\\ 65\\ 0\\ \end{array} \right) , \end{aligned}$$

and suppose the marginal homogeneity CMM in Eq. 9 is the CMM of interest. The ML estimate is $\widehat{\textbf{m}} = (0, 32.5, 0, 32.5, 32.5, 0, 32.5, 0)^\textrm{T}$ with $G^2 = 180.22$ (df = 2). For MEL estimation, the second-order estimation problems occur. Because

20 $$\begin{aligned} \textbf{n}^* = \left( \begin{array}{c} n^{abc}_{100}\\ n^{abc}_{110}\\ \end{array} \right) = \left( \begin{array}{c} 65\\ 65\\ \end{array} \right) , \end{aligned}$$

Eq. 9 reduces to

21 $$\begin{aligned} \textbf{g}^*(\textbf{m}^*) = \left( \begin{array}{rr} 1 &{} 0 \\ 1 &{} 1 \\ \end{array} \right) \left( \begin{array}{c} m^{abc}_{100}\\ m^{abc}_{110}\\ \end{array} \right) = \left( \begin{array}{r} 0\\ 0\\ \end{array} \right) . \end{aligned}$$

The rows of the design matrix in Eq. 21 contain only nonnegative elements, and the constraints imply that $m^{abc}_{100} = m^{abc}_{110} = 0$. But since $n^{abc}_{100} > 0$ and $n^{abc}_{110} > 0$, the likelihood function is zero whenever Eq. 21 holds; that is, $G^2=\infty$.

The problem of a zero likelihood can be circumvented by adding $n^{abc}_{011}$ to $\textbf{n}^*$. Then we obtain

22 $$\begin{aligned} \begin{array}{ccc} \textbf{n}^\dagger = \left( \begin{array}{r} n^{abc}_{011}\\ n^{abc}_{100}\\ n^{abc}_{110}\\ \end{array} \right) = \left( \begin{array}{r} 0\\ 65\\ 65\\ \end{array} \right) &{} \text{ and } \textbf{g}^\dagger (\textbf{m}^\dagger ) = \left( \begin{array}{rrr} -1 &{} 1 &{} 0 \\ -1 &{} 1 &{} 1 \\ \end{array} \right) \left( \begin{array}{c} m^{abc}_{011}\\ m^{abc}_{100}\\ m^{abc}_{110}\\ \end{array} \right) = 0; \end{array} \end{aligned}$$

yielding $\widehat{\textbf{m}}^\dagger = (65, 65, 0)^\textrm{T}$ with $G^2 = 1906.93$ $(df = 2)$. Neither ML nor MAEL fit the data well but $G^2$ is almost 10 times larger for MAEL than for ML. Including more cells may decrease the difference in global fit between MAEL and ML. The second-order estimation problem can be circumvented if $n^{abc}_{000}$, $n^{abc}_{011}$, and $n^{abc}_{101}$ are added to $\textbf{n}^*$. In this way, $\textbf{n}^\dagger$ includes all bivariate margins:

23 $$\begin{aligned} \begin{array}{ccc} \textbf{n}^{\dagger } = \left( \begin{array}{c} n^{abc}_{000}\\ n^{abc}_{011}\\ n^{abc}_{100}\\ n^{abc}_{101}\\ n^{abc}_{110}\\ \end{array} \right) = \left( \begin{array}{r} 0\\ 0\\ 65\\ 0\\ 65\\ \end{array} \right) , &{} \text{ and } &{} \textbf{g}^\dagger (\textbf{m}^\dagger ) = \left( \begin{array}{rrrrr} 0 &{} -1 &{} 1 &{} 1 &{} 0 \\ 0 &{} -1 &{} 1 &{} 0 &{} 1 \\ \end{array} \right) \left( \begin{array}{c} m^{abc}_{000}\\ m^{abc}_{011}\\ m^{abc}_{100}\\ m^{abc}_{101}\\ m^{abc}_{110}\\ \end{array} \right) = 0; \end{array} \end{aligned}$$

yielding $\widehat{\textbf{m}}^\dagger = (0, 54.167, 32.5, 21.67, 21.67)^\textrm{T}$ with $G^2 = 232.92$ (df = 2). $G^2$ is now much closer to $G^2$ of the ML solution.

## Comparing ML, MEL, and MAEL

Two studies compared the ML, MEL, and MAEL estimation procedures for three CMMs relevant for psychology and educational sciences:

1. *Model “Alpha"*. Kuijpers et al. (2013) showed that testing whether Cronbach’s alpha ($\alpha$) equals a certain benchmark can be done using a CMM with 1 degree of freedom. Model “Alpha" is $\alpha =.8$, because .8 is an arbitrary but commonly used benchmark to assess the quality of the test-score reliability (see, e.g., Nunnally, 1978).
2. *Model *“$H_j$". For a set of *J* items, Van der Ark et al. (2008) showed that testing whether each item-scalability coefficients $H_j$ ($j = 1,..., J$) equals the researcher-specified lower-bound values *c* can be done using a CMM with *J* degree of freedom. Let $\textbf{H} = (H_1,..., H_J)^T$. Model “$H_j$” is $\textbf{H} =.3 \, \textbf{1}$, as.3 is the default value of for lower bound *c* provided by software programs for Mokken scale analysis.
3. *Model “Mean”*. Bergsma et al. (2009, pp. 185–188) showed that testing equality of means of *J* variables can be done using a CMM with $J-1$ degrees of freedom. Investigating equality of means may be useful when investigating whether a set of items are parallel (e.g., Lord & Novick, 1968, pp. 47–50)

Study 1 is an exploratory simulation study to investigate the convergence rate and computation time under various settings. The tables are small to allow ML estimation. In Study 2, we investigated the Type I error rate of CMMs estimated with MAEL for realistic numbers of items in psychological and educational test data. We considered tables ranging from small (16 cells) to enormous ($1.1 \times 10^{12}$). In addition, we investigated bias and variance of parameter ${\varvec{\upbeta }}$. ML estimation was not considered because it is feasible only for small tables, and MEL estimation was not considered because in most cases the algorithm runs into singularity problems and, consequently, does not converge.

### Population Models and Estimation

Both Study 1 and Study 2 required population models (i.e., the vector of probabilities, ${{\varvec{\uppi }}}$) that comply with the constraints of the CMM under consideration (i.e., “Model Alpha”, “Model $H_j$”, or “Model Mean” for *J* items). The population models were constructed as follows. First, we constructed a two-parameter logistic model (2PLM), a popular item response theory model (Birnbaum, 1968), for which the location and discrimination parameters were selected (by trial and error) such that data generated from that 2PLM were close, in a loose sense, to the requirements of the CMM under consideration. Next, we generated 1000 response patterns from the 2PLM. Then, using ML (Study 1) or MAEL (Study 2), the CMM under consideration was estimated for the generated data, and the resulting estimated probabilities were used as the probabilities ${{\varvec{\uppi }}}$ of the data generating model. Finally, *N* observations were sampled from ${{\varvec{\uppi }}}$. This data-generating procedure yields expected frequencies $\textbf{m}$ that meet the constraints of the CMM of interest and have a relatively close fit to the 2PLM.

In Study 1, a certain percentage of the probabilities from the population model was deliberately set to zero, so as to create conditions with many zero cells. The cells in ${{\varvec{\uppi }}}$ that were set to zero were randomly selected, and afterwards ${{\varvec{\uppi }}}$ was rescaled. Note that setting random cells to zero is useful to investigate convergence, but makes investigation of Type I error and bias impossible.

The CMMs under consideration were estimated using the generated data as input, employing the R package cmm (Bergsma & Van der Ark, 2023), which offers MAEL estimation starting from version 1.0. All CMMs received uniform starting values and a maximum of 1,000 iterations. The code is available on the Open Science Framework at https://osf.io/yz8rm/).

### Study 1: convergence Rates and Computation Times

For $N = 50$, we investigated the effect of four independent variables on convergence rate and computation time. *Estimation Procedure* had three levels: ML, MEL, and MAEL. *Type of CMM* had three levels: “Model Alpha”, “Model $H_j$”, and “Model Mean”. For “Model Alpha” the criterion value was set to the sample value plus. 2; and for “Model $H_j$” the criterion value was set to the average of the sample $H_j$ values. For convenience, the criterion values depend on the sample values. Because Study 1 investigated only computation time and convergence rate, sample-dependent criterion values are not a problem. *Minimum Percentage Cells with Zero Observed Frequency* (*U*) had three levels: 0% (none), 25% (small percentage), and 75% (large percentage). *Number of items* (*J*) had two levels: 4 dichotomous items, yielding $L = 16$ possible response patterns, and 8 items, yielding $L = 256$ response patterns. The number of items was kept small to allow for ML estimation. Hence, we had a 3 (Estimation Method) $\times$ 3 (CMM) $\times$ 3 (*U*) $\times$ 2 (*J*) experimental design with a total of 54 cells. Each cell in the experimental design was replicated 1,000 times. For a small extra design (100 replications), we estimated CMMs with 10 ($L = 1024$) items to demonstrate the sharp increase in computation time.

Table 1 shows that for the smallest tables ($J = 4$ and $J = 8$), both ML and MAEL almost always converged, whereas MEL often broke down for models “$H_j$” and “Mean”. For $J = 10$, ML ran into memory problems for models “$H_j$” and “Mean”, whereas MEL almost always broke down. For Model “Alpha”, convergence results were satisfactory for all three estimation methods.

**Table 1:** Convergence rates (percentage) and median computation times in seconds for ML, MEL, and MAEL, for three different CMMs, two numbers of items (*J*), and three percentages of unobservable response patterns (*U*) based on $1,\!000$ ($J = 4, 8$) and 100 (${ J} = 10$) replications.

CMM	*J*	*U*	Convergence rate				Median computation time		
			ML	MEL	MAEL		ML	MEL	MAEL
Alpha	4	0%	100.0	100.0	100.0		0.00	0.00	0.00
		25%	100.0	100.0	100.0		0.01	0.00	0.01
		75%	100.0	96.6	100.0		0.00	0.00	0.00
	8	0%	100.0	100.0	100.0		1.14	0.00	0.05
		25%	100.0	100.0	100.0		1.16	0.00	0.06
		75%	100.0	100.0	100.0		1.25	0.00	0.09
	10	0%	100.0	100.0	100.0		107.26	0.10	0.30
		25%	100.0	100.0	100.0		114.13	0.05	0.39
		75%	100.0	100.0	100.0		108.56	0.10	0.48
$H_j$	4	0%	100.0	75.5	99.5		0.00	0.00	0.00
		25%	100.0	75.6	99.5		0.00	0.00	0.00
		75%	100.0	0.0	100.0		0.00	NA	0.00
	8	0%	99.8	47.6	99.5		0.14	0.02	0.01
		25%	100.0	52.8	99.8		0.14	0.03	0.01
		75%	99.7	40.2	99.7		0.14	0.82	0.02
	10	0%	0.0	36.0	99.0		NA	0.78	0.50
		25%	0.0	30.0	98.0		NA	1.13	0.60
		75%	0.0	31.1	99.0		NA	1.12	0.80
Mean	4	0%	100.0	45.9	100.0		0.01	0.01	0.01
		25%	100.0	20.8	100.0		0.01	0.01	0.01
		75%	100.0	0.0	100.0		0.01	NA	0.01
	8	0%	100.0	2.9	100.0		0.02	0.02	0.02
		25%	100.0	3.1	100.0		0.02	0.02	0.02
		75%	100.0	0.5	100.0		0.02	0.02	0.02
	10	0%	0.0	2.0	100.0		NA	0.37	0.02
		25%	0.0	0.0	100.0		NA	NA	0.03
		75%	0.0	0.0	100.0		NA	NA	0.03

The distribution of the computation time was positively skewed. Therefore, we reported the median rather than the mean computation time. Naturally, MAEL and MEL were at least as fast as ML: Ranging from just as fast to more than 200 times faster. As the number of items increased, the computation time increased dramatically (Table 1, columns 4–6). This was especially true for ML estimation. For 4 and 8 items ($L=256$), but the computation time was still reasonable in all sample (never longer than 100 s), but for 10 items ($L=1024$) some runs took up to 30 min for Model “Alpha”.

The results show that even for moderately large tables, ML may run into memory problems. Moreover, the results show that the first- and second-order estimation problems are omnipresent so that MEL often breaks down. This leaves MAEL as the viable candidate for estimating CMMs for large sparse contingency tables.

### Study 2: Type I Error Rate

For MAEL estimation, we investigated the effect of the type of CMM, the number of items, and sample size on the Type I error rate and the bias and standard deviation of model parameter ${\varvec{\upbeta }}$. (Eq. 2). As in Study 1, Type of CMMs had three levels: “Model Alpha” (the criterion value was set to 0.8), “Model $H_j$” (the criterion value was set to 0.3), and “Model Mean”. For “Model Alpha” and “Model $H_j$” parameter ${\varvec{\upbeta }}$ is fixed to $\beta =0.8$ and ${\varvec{\upbeta }}=\textbf{1}_J \cdot 0.3$, respectively. Hence, bias and standard deviation of ${\varvec{\upbeta }}$ were investigated only for Model “Mean”, where $\beta$ equals the overall mean item score. Moreover, we studied four levels of number of items: 4 ($L = 16$), 8 ($L = 256$), 20 ($L = 1,048,576$), and 40 ($L \approx 1.1 \times 10^{12}$); and three levels of sample size ($N = 250$, $N = 500$, and $N=1000$). Hence, we had a $3 \text{(CMM) } \times 4~(J)~\times 3~(N)~$ experimental design with a total of 36 cells. Each cell in the experimental design was replicated 10,000 times for $J = 4$ and $J = 8$ items and 1000 times for $J = 20$ and $J = 40$ items. The empirical Type I error rate over the replications was compared to the nominal Type I error rate of 0.05, the mean value of $\hat{\beta } - \beta$ over replications was used to estimate the bias, and the standard deviation of $\hat{\beta }$ over replications was used as an estimate of the standard error of $\hat{\beta }$.

**Fig. 1:**
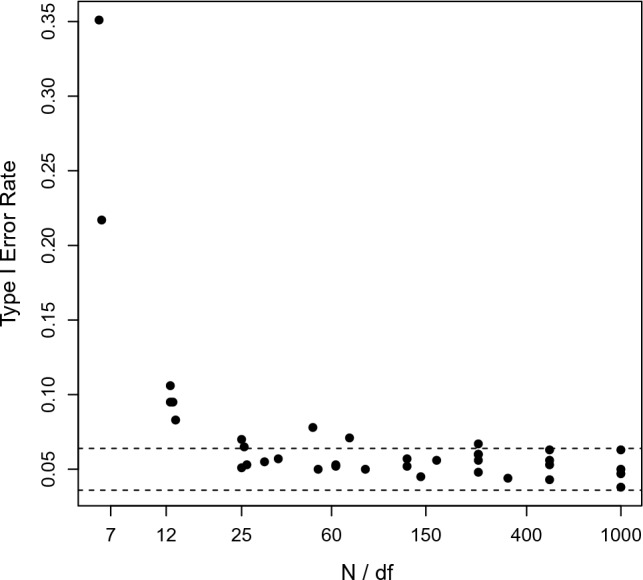
Type I error rates by the ratio of sample size and degrees of freedom in Study 2. Dashed lines are the limits of the 95% confidence interval of the Type I error rate due to Monte Carlo error.

Table 2 shows the Type I error rates for all cells in the design. In most cells, the Type I error rates are close to the nominal Type I error rate. For models with many degrees of freedom estimated using a relatively small sample size, the models are too liberal. For 40 items, models “$H_j$” and “Mean” have 40 and 39 degrees of freedom, respectively. For $N=250$, this results in approximately 6 observations per degree of freedom. Hence, the poor performance is not so much due to the large table as due to the increase in degrees of freedom. Results are satisfactory if the sample size per degree of freedom exceeds 25 (see Fig. 1).

For Model “Mean”, the bias of ${\varvec{\upbeta }}$ (not tabulated) was negligible in all cases, and the estimated standard error (Table 3) behaved as expected; that is, if *N* doubles, the estimated standard error decreased approximately by a factor $\sqrt{2}$.

**Table 2:** Type I error rate for MAEL estimation of three different CMMs, four different numbers of items (*J*), and three different sample sizes (*N*), based on 1000 replications.

Model	$\textit{df}$	*N*	*J*			
			4	8	20	40
Alpha	1	250	0.048	0.060	0.056	0.060
		500	0.053	0.063	0.056	0.043
		1000	0.063	0.050	0.047	0.038
$H_j$	*J*	250	0.052	0.055	**0.095**	**0.351**
		500	0.057	0.053	0.051	**0.106**
		1000	0.067	0.052	**0.078**	**0.070**
Mean	$J-1$	250	0.048	0.052	0.056	0.053
		500	0.048	0.054	0.050	0.055
		1000	0.046	0.049	0.055	0.050

*Note*: A 95% confidence interval for the Type I error rate equals [0.036;0.064]. Values outside the 95% confidence interval are printed in boldface.

**Table 3:** Estimated standard error of CMM-parameter estimate $\hat{\beta }$ for Model “Mean”, for four different numbers of items (*J*), and three different sample sizes(*N*), based on 1000 ($J= 20$ and $J=40$) and 10, 000 ($J= 4$ and $J=8$) replications.

Model	$\textit{df}$	*N*	*J*			
			4	8	20	40
Mean	$J-1$	250	0.020	0.018	0.003	0.002
		500	0.014	0.013	0.003	0.002
		1000	0.010	0.009	0.002	0.001

## Discussion

CMMs have potential for application to psychological data, but an important reason that this potential has so far not been realized may be that up to now ML estimation of CMMs could only be applied to contingency tables for a limited number of categorical variables (up to, say, 10–20 variables, depending on the number of categories per variable). The present paper shows that this limitation can be resolved by the newly introduced maximum augmented empirical likelihood (MAEL) estimation method, a procedure that considers all nonzero cells in the table (i.e., cells with at least one observation) and some well-chosen zero cells in the table (i.e., cells with no observations). MAEL can be thought of as lying in between maximum empirical likelihood (MEL) estimation, which considers only nonzero cells in the table and subsequently suffers from the first-order and second-order estimation problems, and maximum likelihood (ML), which considers all cells in the table and runs into memory problems if the table is large.

The asymptotic distribution of the ML estimators of marginal parameters is known (Lang 2005), and depends only on the covariance matrix of the sample marginal distributions. In contrast to MEL, due to the augmentation step MAEL allows this covariance matrix to be estimated. Simulation study 2 shows this estimation is done sufficiently well in a number of practical settings, in particular, the asymptotic distribution of the ML estimators also provide a good approximation of the distribution of the MAEL estimators. The asymptotic distributions of ML and MAEL estimators are identical.

MAEL estimation has advantages compared to alternative methods which can be used to estimate CMMs for large contingency table, namely the weighted least squares method (Grizzle et al., 1969, a.k.a. the GSK-method), generalized estimating equations (GEEs, e.g., Qaqish & Liang, 1992), and composite likelihood (e.g., Varin et al., 2011). A comparison of GSK and GEE with ML estimation is given in Rudas and Bergsma (2023). All these four methods can be used to estimate CMMs for almost arbitrarily large contingency tables, but the only methods with guaranteed optimal asymptotic efficiency are MAEL and GSK. Unlike MAEL, however, GSK is sensitive to sparsity of the marginal distributions (Bergsma et al., 2013, see also the discussion of Berkson, 1980).

Like GEE and GSK, MAEL estimation is computationally fast, and like ML but unlike GEE, it is asymptotically efficient. Furthermore, MAEL is less sensitive to sparsity of the marginal distributions than GSK. Thus, MAEL seems to be the preferred method for estimating CMMs. Researchers should take heed that if the ratio of the sample size and degrees of freedom becomes too small (say less than 25), the Type I error rates may be too liberal. This is not a feature of MAEL per se, but for all models that are too complex for the number of observations. Composite likelihood estimation is a possibly attractive alternative for estimating CMMs, which was not considered in this study because the estimation procedures are not yet available for CMMs, whereas MAEL fits nicely in the ML framework and software that is already available for CMMs. In addition, composite likelihood is a quasi-likelihood method, and hence asymptotic efficiency is lost, whereas ML, and hence MAEL and MEL, are asymptotically efficient (Aitchison & Silvey 1958, Lang, 2005).

## Appendix A: First- and Second-Order Estimation Problems

With $\textbf{X}$ a random variable, MEL can be used to make inferences on a Euclidean parameter ${{\varvec{\uptheta }}}$ of the distribution of $\textbf{X}$, where ${{\varvec{\uptheta }}}$ is defined by an estimating equation of the form

24 $$\begin{aligned} E{{\varvec{\uppsi }}}(\textbf{X},{{\varvec{\uptheta }}})=0 \end{aligned}$$

for some function ${{\varvec{\uppsi }}}$. For example, if ${{\varvec{\uppsi }}}(\textbf{x},{{\varvec{\uptheta }}})=\textbf{x}-{{\varvec{\uptheta }}}$, then (24) implies that ${{\varvec{\uptheta }}}=E\textbf{X}$. Denote the population value of ${{\varvec{\uptheta }}}$ by ${{\varvec{\uptheta }}}_0$. Suppose we have observed $\textbf{x}_1,\ldots ,\textbf{x}_N$, which are i.i.d. and distributed as $\textbf{X}$. The MEL estimator ${\hat{{{\varvec{\uptheta }}}}}$ of ${{\varvec{\uptheta }}}$ defined by (24) solves the constrained optimization problem

$$\begin{aligned} \max _{{{\varvec{\uptheta }}},{{\varvec{\uppi }}}}\prod _{i=1}^n\pi _i \end{aligned}$$

subject to

25 $$\begin{aligned} \pi _i\ge 0, \sum \pi _i=1,\sum \pi _i{{\varvec{\uppsi }}}(\textbf{x}_i,{{\varvec{\uptheta }}})=0 \end{aligned}$$

The first-order estimation problem occurs if (25) does not have a solution (This problem is also known as the *empty set problem*, see Grendár & Judge, 2009). The best known example is the case that ${{\varvec{\uptheta }}}=E\textbf{X}$ while the population mean lies outside the convex hull of $\{\textbf{x}_1,\ldots ,\textbf{x}_N\}$ (Qin and Lawless, 1994).

Let *F* be the distribution function of $\textbf{X}$. Under some conditions on ${{\varvec{\uppsi }}}$ and *F*, ${\hat{{{\varvec{\uptheta }}}}}$ has an asymptotic multivariate normal distribution, in particular,

$$\begin{aligned} \sqrt{n}({\hat{{{\varvec{\uptheta }}}}}-{{\varvec{\uptheta }}}_0) \sim \text{ MVN }(\textbf{0},\textbf{V}_F({{\varvec{\uptheta }}}_0)) \end{aligned}$$

where

$$\begin{aligned} \textbf{V}_F({{\varvec{\uptheta }}}) = {{\varvec{\Psi }}}_F({{\varvec{\uptheta }}})^{-1}\textbf{W}_F({{\varvec{\uptheta }}}){{\varvec{\Psi }}}_F({{\varvec{\uptheta }}})^{-1} \end{aligned}$$

and

$$\begin{aligned} {{\varvec{\Psi }}}_F({{\varvec{\uptheta }}}) = \int \left( \frac{d{{\varvec{\uppsi }}}(\textbf{x},{{\varvec{\uptheta }}})}{d{{\varvec{\uptheta }}}^T}\right) dF(\textbf{x}) \hspace{10mm}\textbf{W}_F({{\varvec{\uptheta }}}) = \int {{\varvec{\uppsi }}}(\textbf{x},{{\varvec{\uptheta }}}){{\varvec{\uppsi }}}(\textbf{x},{{\varvec{\uptheta }}})^TdF(\textbf{x}) \end{aligned}$$

The second-order estimation problem occurs if there does not exist a distribution function *G* with empirical support $\{\textbf{x}_1,\ldots ,\textbf{x}_N\}$ such that $\textbf{V}_F({{\varvec{\uptheta }}}_0)=\textbf{V}_G({{\varvec{\uptheta }}}_0)$.

### Example 7

To illustrate the second-order estimation problem, consider a $2\times 2$ contingency table with cell probabilities $\pi _{ij}>0$ ($i,j\in \{0,1\}$), and let $\theta$ be the log ratio of marginal odds; that is,

$$\begin{aligned} \theta =\log \frac{\pi _{1+}/\pi _{2+}}{\pi _{+1}/\pi _{+2}} \end{aligned}$$

(For details on how to define ${{\varvec{\uppsi }}}$ such that this $\theta$ is the solution of (24), see Owen, 2001). If one observed marginal count is zero, then $\theta =\pm \infty$ under empirical likelihood; that is, the first-order estimation problem occurs since $-\infty<\theta _0<\infty$. If the two observed off-diagonal cell counts are zero, then $\theta =0$ under empirical likelihood and as a consequence $V_G(\theta )=0$ for any distribution *G* with support the two diagonal cells. However, assuming no structural zeroes in the table, $V_F(\theta _0)>0$, and therefore the second-order estimation problem occurs. In this case, the first-order estimation problem occurs in addition to the second-order one if $\theta _0\ne 0$.

A special case of the second-order estimation problem has been identified earlier by Bergsma et al. (2012), who called it the *zero-likelihood problem*. It occurs if the empirical likelihood is zero for all solutions of (25). In this case, for any distribution *G* with support $\{\textbf{x}_1,\ldots ,\textbf{x}_N\}$, $\textbf{V}_G({{\varvec{\uptheta }}})$ is a matrix of zeroes, unequal to $\textbf{V}_F({{\varvec{\uptheta }}}_0)$; hence, the second-order estimation problem occurs.

We propose a solution for both estimation problems by augmentation of the support of the empirical likelihood, resulting in an estimation procedure lying in a spectrum with ML at one extreme and MEL at the other, which we call *maximum augmented empirical likelihood* (MAEL) estimation.

## B Algorithm for Maximum Likelihood Estimation

Under some regularity conditions, the maximum likelihood estimates under model (5) are a saddle point of the Lagrangian log-likelihood

26 $$\begin{aligned} L(\textbf{m},\mu ,{{\varvec{\lambda }}}) = \textbf{n}^\textrm{T} \log (\textbf{m}) - \mu (\textbf{1}^\textrm{T} \textbf{m} - N) + {{\varvec{\lambda }}}^\textrm{T} \textbf{g}(\textbf{m}). \end{aligned}$$

where $\mu$ and ${{\varvec{\lambda }}}$ are Lagrange multipliers. In Eq. 26, $\textbf{n}^\textrm{T} \log (\textbf{m})$ is the unconstrained kernel of the log-likelihood, and the Lagrangian terms are added to satisfy the multinomial sampling constraint $\sum _i m_i = N$ (Eq. 14) and the model constraint (Eq. 13). Bergsma (1997, pp. 89–95) developed a Fisher scoring algorithm to find the ML estimates of the constrained expected frequencies in $\textbf{m}$ (Eq. 5) or, equivalently, the constrained cell probabilities ${{\varvec{\uppi }}}$. This algorithm is a modification of Lagrangian algorithms by Aitchison and Silvey (1958) and Lang and Agresti (1994).

It can be shown that $\mu = 1$, so Eq. 26 can be simplified to

27 $$\begin{aligned} L(\textbf{m},{{\varvec{\lambda }}}) = \textbf{n}^\textrm{T} \log (\textbf{m}) + {{\varvec{\lambda }}}^\textrm{T} \textbf{B}^\textrm{T}\textbf{g}(\textbf{A}^\textrm{T} \textbf{m}). \end{aligned}$$

The ML estimates of the $\textbf{m}$ and ${{\varvec{\lambda }}}$ are obtained by means of an iterative procedure that determines a saddle point of this Lagrangian.

We take the derivative of $L(\textbf{m},{{\varvec{\lambda }}})$ with respect to $\log \textbf{m}$ rather than $\textbf{m}$ because they yield simpler expressions. Note that $\partial L(\textbf{m},{{\varvec{\lambda }}})/ \partial \log \textbf{m} = 0$ iff $\partial L(\textbf{m},{{\varvec{\lambda }}})/ \partial \textbf{m} = 0$. Let $\textbf{G} = \textbf{G}(\textbf{m})$ be the Jacobian of $\textbf{g}(\textbf{m})$ with respect to $\log \textbf{m}$. Differentiating $L(\textbf{m},{{\varvec{\lambda }}})$ with respect to $\log (\textbf{m})$ yields

$$\begin{aligned} \textbf{l}(\textbf{m},{{\varvec{\lambda }}}) = \textbf{n} - \textbf{m} + \textbf{G}{{\varvec{\lambda }}}. \end{aligned}$$

Under suitable regularity conditions, the ML estimator $\widehat{\textbf{m}}$ is a vector $\textbf{m}$ for which there is a Lagrange multiplier vector ${{\varvec{\lambda }}}$ such that the simultaneous equations

$$\begin{aligned} \textbf{l}(\textbf{m},{{\varvec{\lambda }}})= & {} 0 \end{aligned}$$

and

$$\begin{aligned} \textbf{g}(\textbf{m})= & {} 0 \end{aligned}$$

are satisfied. Then, the *expected value* of the derivative matrix of the vector $(\textbf{l}(\textbf{m},{{\varvec{\lambda }}}),\textbf{g}(\textbf{m}))$ with respect to $( \log \textbf{m},{{\varvec{\lambda }}})$ is

$$\begin{aligned} \textbf{V}(\textbf{m}) = \left( \begin{array}{cc} E\left( \frac{\displaystyle \partial \textbf{l}(\textbf{m},{{\varvec{\lambda }}})}{\displaystyle \partial \log \textbf{m}^\textrm{T} } \right) &{} E\left( \frac{\displaystyle \partial \textbf{g}(\textbf{m})}{\displaystyle \partial \log \textbf{m}^\textrm{T} } \right) \\ E\left( \frac{\displaystyle \partial \textbf{l}(\textbf{m},{{\varvec{\lambda }}})}{\displaystyle \partial {{\varvec{\lambda }}}^\textrm{T} } \right) &{} E\left( \frac{\displaystyle \partial \textbf{g}(\textbf{m})}{\displaystyle \partial {{\varvec{\lambda }}}^\textrm{T} } \right) \end{array} \right) = \left( \begin{array}{cc} -\textbf{D}(\textbf{m}) &{} \textbf{G} \\ \textbf{G}^\textrm{T} &{} 0 \end{array} \right) . \end{aligned}$$

Let $\textbf{n}^+$ be equal to the vector $\textbf{n}$ with zeroes replaced by a small positive constant (say, $10^{-10}$), and define the Fisher scoring starting values

$$\begin{aligned} \left( \begin{array}{c} \log \textbf{m}^{(0)} \\ {{\varvec{\lambda }}}^{(0)} \end{array} \right)= & {} \left( \begin{array}{c} \log \textbf{n}^+ \\ 0 \end{array} \right) \end{aligned}$$

and, for $$k=0,1,\ldots $$,

$$\begin{aligned} \left( \begin{array}{c} \log \textbf{m}^{(k+1)} \\ {{\varvec{\lambda }}}^{(k+1)}) \end{array} \right)= & {} \left( \begin{array}{c} \log \textbf{m}^{(k)} \\ {{\varvec{\lambda }}}^{(k)}) \end{array} \right) - \textbf{V}(\textbf{m}^{(k)})^{-1} \cdot \left( \begin{array}{c} \textbf{l}(\textbf{m}^{(k)},{{\varvec{\lambda }}}^{(k)}) \\ \textbf{g}(\textbf{m}^{(k)}) \end{array} \right) . \ \end{aligned}$$

Then, as $k\rightarrow \infty$, $\textbf{m}^{(k)}$ should go to $\widehat{\textbf{m}}$. Tedious but straightforward matrix algebra yields the simplified form

$$\begin{aligned} \log \textbf{m}^{(k+1)}= & {} \log \textbf{m}^{(k)} + \textbf{D}(\textbf{m}^{(k)})^{-1}\textbf{l}(\textbf{m}^{(k)},{{\varvec{\lambda }}}^{(k+1)}) \\ {{\varvec{\lambda }}}^{(k+1)}= & {} - (\textbf{G}^\textrm{T} \textbf{D}(\textbf{m}^{(k)})\textbf{G})^{-1}(\textbf{G}^\textrm{T} \textbf{D}(\textbf{m}^{(k)})^{-1}(\textbf{n}-\textbf{m}^{(k)})+\textbf{g}(\textbf{m}^{(k)}). \end{aligned}$$

This algorithm does not always converge, and it can be helpful to introduce a step size $\text{ step}^{(k)}\in \langle 0,1]$ as follows:

28 $$\begin{aligned} \log \textbf{m}^{(k+1)} = \log \textbf{m}^{(k)} + \text{ step}^{(k)}\textbf{D}(\textbf{m}^{(k)})^{-1}\textbf{l}(\textbf{m}^{(k)},{{\varvec{\lambda }}}^{(k+1)}) \end{aligned}$$

Note that the update of ${{\varvec{\lambda }}}$ is left unchanged.

The step size should be chosen so that the new estimate $\textbf{m}^{(k+1)}$ is better than the old estimate $\textbf{m}^{(k)}$. A criterion for deciding this is obtained by defining the following quadratic form measuring the distance from convergence:

$$\begin{aligned} \delta (\textbf{m}^{(k)}) = \textbf{l}(\textbf{m}^{(k)},{{\varvec{\lambda }}}^{(k+1)})\textbf{D}(\textbf{m}^{(k)})^{-1}\textbf{l}(\textbf{m}^{(k)},{{\varvec{\lambda }}}^{(k+1)}). \end{aligned}$$

Convergence is reached at $\textbf{m}$ if and only if $\delta (\textbf{m})=0$ and therefore, if possible, the step size should be chosen so that $\delta (\textbf{m}^{(k+1)})< \delta (\textbf{m}^{(k)})$ for all *k*. This is possible if the tentative solution is sufficiently close to the ML estimate. Otherwise, a recommendation which seems to work very well in practice is to jump to another region by taking a step size equal to one.
